# Gonadal Transcriptome Analysis of Male and Female Olive Flounder (*Paralichthys olivaceus*)

**DOI:** 10.1155/2014/291067

**Published:** 2014-07-06

**Authors:** Zhaofei Fan, Feng You, Lijuan Wang, Shenda Weng, Zhihao Wu, Jinwei Hu, Yuxia Zou, Xungang Tan, Peijun Zhang

**Affiliations:** ^1^Key Laboratory of Experimental Marine Biology, Institute of Oceanology, Chinese Academy of Sciences, 7 Nanhai Road, Qingdao 266071, China; ^2^University of the Chinese Academy of Sciences, Beijing 10049, China

## Abstract

Olive flounder (*Paralichthys olivaceus*) is an important commercially cultured marine flatfish in China, Korea, and Japan, of which female grows faster than male. In order to explore the molecular mechanism of flounder sex determination and development, we used RNA-seq technology to investigate transcriptomes of flounder gonads. This produced 22,253,217 and 19,777,841 qualified reads from ovary and testes, which were jointly assembled into 97,233 contigs. Among them, 23,223 contigs were mapped to known genes, of which 2,193 were predicted to be differentially expressed in ovary and 887 in testes. According to annotation information, several sex-related biological pathways including ovarian steroidogenesis and estrogen signaling pathways were firstly found in flounder. The dimorphic expression of overall sex-related genes provides further insights into sex determination and gonadal development. Our study also provides an archive for further studies of molecular mechanism of fish sex determination.

## 1. Introduction

According to Helfman et al., there are almost 30,000 species of fish distributed nearly in all the aquatic habitats around the world [1]. They are the most abundant vertebrates on Earth, showing a diversity of species unmatched by other classes. Not surprisingly given this extreme diversity, fish exhibit all known forms of vertebrate sex determination to adapt to the variable habitats [2]. In the meantime, economic values of growth rate, time and age of maturation, body shape, and carcass composition are related to their sexual development in some edible fish species [3]. Then, we are interested in fish sex determination mechanism. As reported, fish sex determination patterns can be classified as genetic sex determination (GSD) and environmental sex determination (ESD) forms. However, the feature of sex determination in fish is remarkably flexible; even individuals with GSD can be influenced by environmental factors like temperature, that is, GSD + EE (environmental effects) form [4]. Although fish have several different sex determination forms, it is hypothesized that genes involved in sex determination are probably conserved throughout evolution. Several genes have been confirmed as master genes of sex determination in some fish species. In the medaka (*Oryzias latipes*), a homologue of the* dmrt1* gene (called* dmy*) is located on the Y chromosome, and its expression is a necessary and sufficient condition for triggering testicular development in bipotential gonads [5, 6]. Recently, five novel sex determining genes (or candidates) have been reported in other fish:* amhy* in Patagonian pejerrey (*Odontesthes hatcheri*) [7],* irf9y* in rainbow trout (*Oncorhynchus mykiss*) [8],* gsdf* in* Oryzias luzonensis* (a relative of medaka) [9],* amhr2* in fugu (*Takifugu rubripes*) [10], and* dmrt1* in half-smooth tongue sole (*Cynoglossus semilaevis*) [11]. Besides the sex determining genes, some conserved genes shown to play important roles in mammal sex determination and differentiation were cloned and identified in fish. These include* cyp19*,* foxl2*,* sf1*,* dax1*,* wt1*,* mis*,* dmrt*, and* sox9* [12]. In mammals, these genes act together to constitute complicated network whereby sex phenotype is established [13]. However, studies on the function and connections of the above genes in fish are limited. More or novel sex-related genes are also needed to be found out. And then, the complex mechanism of fish sex determination could be adequately explained.

Over the past decade, significant progress has been made in genome-wide gene expression profiling by the development and application of large scale sequencing technique, which can easily show more differential expressional genes in different traits, such as gender. Transcriptome profiling associated with sex determination and differentiation using RNA-seq of several fish, including platyfish (*Xiphophorus maculatus*) [14], rainbow trout (*O. mykiss*) [15], Nile tilapia (*Oreochromis niloticus*) [16], rockfish (*Sebastiscus marmoratus*) [17], catfish (*Ictalurus punctatus*) [18], and turbot (*Scophthalmus maximus*) [19], was shown. These data provided transcriptomic information expressed in gonads at particular condition and time and identified sex differentially expressed genes, while there is almost no report on biological pathways including gonadal steroidogenesis pathway in male and female fish.

As an important commercially marine flatfish, olive flounder (*Paralichthys olivaceus*) is mainly cultured in China, Japan, and Korea. The female flounder grows significantly faster and bigger than the male one [20, 21]. Breeding and culturing all-female population of flounder is a promising approach to boost production. Thus, the study on sex manipulation of flounder has been attracting researchers' interests. Olive flounder has XX (female)/XY (male) sex determination system indirectly inferred from the female-dominant phenotype among the gynogenesis offspring [21]. Its formation of sex phenotype is also influenced by environmental factors such as water temperature and external hormone [22]. Sex-related conserved genes have been studied to elucidate the sexual molecular mechanism in flounder. Kitano et al. firstly cloned flounder* cyp19a* gene and found female predominant expression pattern [23], whereafter cloning and expression profile analysis of* cyp19a* and its transcription factors such as* foxl2* [24] and* dmrt1 *[25] and other sex-related genes including* cyp17* [26] and* dmrt4 *[27] were conducted with male and female flounder. However, we have got only limited message about flounder sex determination, and more sex-related genes and their functions need to be studied. Further identification of the expression profile of genes involved in gonadal development using RNA-seq may help to illuminate the gene regulatory network controlling sex determination and subsequent maintenance of phenotypic sex. In this study, we sequenced flounder gonadal transcriptomes and identified the differences in gene expression profiles between ovary and testis and relevant biological pathways. These data would provide a useful genomic resource for future study on sex determination and for selection of candidate genes involved in these processes in flounder.

## 2. Materials and Methods

### 2.1. Fish

Adult and juvenile flounder (12~40 cm in total length, TL) used in the present study were collected from Shenghang fish farm (Weihai, China) or purchased from Nanshan market (Qingdao, China) and were temporarily cultured in a 3 m^3^ aerated seawater tank at the institute aquarium and fed with commercial particle food twice a day. Gonads were retrieved from the abdomen of fish after anesthetization. Their genders were identified by morphological observation of gonads [28]. Each gonadal sample was divided into two halves. The first half was fixed in Davison's fixative solution for identification of gonadal developmental stage using a histology method as described below. The second half was stored immediately in liquid nitrogen for RNA isolation. Totally, the gonadal tissues at developmental stage I (3 testes and 3 ovaries), II (2 testes and 3 ovaries), and III (2 testes and 3 ovaries) were used in this project. Semen and eggs were gently squeezed out from 2 mature male flounder (40~50 cm in TL) and 3 mature female flounder (50~60 cm in TL), respectively, and the samples were immediately frozen and stored in liquid nitrogen for RNA isolation. All animal work has been conducted according to relevant national and international guidelines. Animal protocols were approved by the Institute of Oceanology, Chinese Academy of Science.

### 2.2. Gonadal Histology

For histological analysis, ovary or testes tissue from each adult or juvenile flounder was fixed in Davison fixative solution for 24 h and stored in 70% ethanol. Pieces of gonadal tissue were cut down, dehydrated using ethanol (gradient: 70%~100%), and finally embedded in paraffin. The sections were conducted at the thickness of 5~7 *µ*m, dewaxed by ethanol (gradient: 100%~50%), washed by distilled water, stained by hematoxylin-eosin, and observed microscopically after air drying. Slices of each sample were mounted on glass slides, stained with hematoxylin, and counterstained with eosin (HE staining) to determine the developmental stage of gonads. Detailed methods can be found in Sun et al. [22] and Radonic and Macchi [29].

### 2.3. RNA Isolation and cDNA Library Construction

Total RNA was isolated from each sample using Trizol Reagent (Invitrogen, USA, http://www.lifetechnologies.com/cn/zh/home/brands/invitrogen) based on the protocol. The genomic DNA was eliminated by treatment with DNase1 (10 U/mL, Ambion, USA, http://www.lifetechnologies.com/cn/zh/home/brands/ambion) at 37°C for 1 h. The purified mRNA was enriched by Micropoly(A) Purist RNA purification kit (Amion, USA), and the concentration and integrity of mRNA were qualified using Agilent 2100 Bioanalyzer (Agilent Technologies, USA, http://www.home.agilent.com). The mRNAs from ovarian developmental stages I, II, and III and egg were mixed together to synthesize cDNA, and mRNAs from testicular developmental stages I, II, and III and sperm were mixed together correspondingly. These blended mRNAs served as templates to synthesize first-strand cDNA using GsuI-oligo dT primer; the reaction was performed with Superscript II reverse transcriptase (Invitrogen, USA) at 42°C for 1 h. Biotins were subsequently attached to the 5′cap of mRNA oxidized by NaIO_4_ (Sigma, USA, http://www.sigmaaldrich.com), whereby the biotin-labeled mRNA /cDNA could be sublimed by Dynal M280 magnetic beads (Invitrogen, USA). Released from the hybrid strands by alkaline lysis, the first-strand cDNA was attached with an adaptor at its 5′end. Second-strand cDNA was synthesized using Ex Taq polymerase (Takara, Japan, http://www.takara-bio.com/) based on the first-strand modified cDNA; then poly(A) and 5′adaptor were trimmed by GsuI enzyme. The synthesized cDNA was disrupted into short fragments (300–500 nt) by ultrasound instrument which were further enriched by Ampure beads (Agencourt, USA, https://www.beckmancoulter.com). These purified cDNA fragments were used to construct cDNA library with the method of TruSeqTM DNA sample Prep kit-set (Illumina, USA, http://www.illumina.com/). Finally, the two cDNA libraries were sequenced on Illumina Solexa using paired-end strategy in a single run.

### 2.4. Illumina Sequencing, Functional Annotation, and Bioinformatics Analysis

Total reads were produced through Illumina Solexa instrument (2 ∗ 100 bp pair-end sequencing) from Chinese National Human Genome Center in Shanghai. The clean reads were obtained from original data by filtering out reads inclusive of unknown nucleotides and low-quality reads in which Q5 percentage (Q5 percentage is proportion of nucleotides with quality value larger than 5) is less than 50%. All clean reads of the two libraries were jointly assembled into contigs performed by Trinity software. The assembled contigs were conducted to predict protein-coding region by GetORF module of EMBOSS package [30]. All the protein-coding sequences were submitted for blastp similarity searches against the NCBI nonredundant (NR) protein database and Eukaryotic Ortholog Groups (KOG) database with the *e*-value of top hit lower than 1 *e* ^−5^. Furthermore, GoPipe software was used to perform blastp (cut-off *e*-value of <1 *e* ^−5^) search against the Swiss-Prot database and TrEMBL database. With the result of blastp, gene ontology (GO) annotation associated with “biological process,” “molecular function,” and “cellular component” was obtained using the gene2go. Likewise, the predicted protein sequence was submitted for bidirectional blastp (cut-off *e*-value of <1 *e* ^−3^) similarity searches against Kyoto Encyclopedia of Genes and Genomes (KEGG) database to assign KEGG Orthology (KO) number. According to the KO assignment, metabolic pathways were generated with tools supplied by KEGG [31]. The mapped read count of given gene is affected by its length and sequencing depth; the reads per kb per million reads (RPKM) were calculated to standardize gene expression level [32]:

(1) $$\begin{array}{c} \text{RPKM}=\frac{10^{6}C}{NL/10^{3}}. \end{array}$$

Here, *C* indicates the mapped read count of a given gene from a given library. *L* indicates the length of a given gene. *N* indicates total mapped read count of a given library.

### 2.5. Identification of Sex-Related Differentially Expressed Genes

RPKM was directly used to compare the difference of gene expression level between male and female. This process was completed by DEGseq (an *R* package) based on the MARS model (MA-plot-based with random sampling model) [33]. We used Benjamini-Hochberg method to determine the threshold of the *q*-value in multiple testing. In our study, “*q* < 10^−3^” and “|log_2 _(RPKM_XX/RPKM_XY)| ≥2” were chosen to identify sex-biased genes. Furthermore, we adopted four strategies to excavate sex-related genes from them: (i) complete cDNA sequences of well-known sex-related genes were downloaded from NCBI nucleotide database (http://www.ncbi.nlm.nih.gov/nucleotide/) and were conducted local blast (cut-off *e*-value of <1 *e* ^−10^) search against the local contigs database; (ii) sex-related keywords were retrieved in the annotation of sex-biased genes; (iii) among the sex-related KEGG pathways, the particular genes encoding pivotal enzyme were selected; (iv) genes reported to be relevant to sex differentiation were selected from the sex-biased genes.

## 3. Results

### 3.1. Assembly, Annotation, and Bioinformatical Analysis

**Figure 1:**
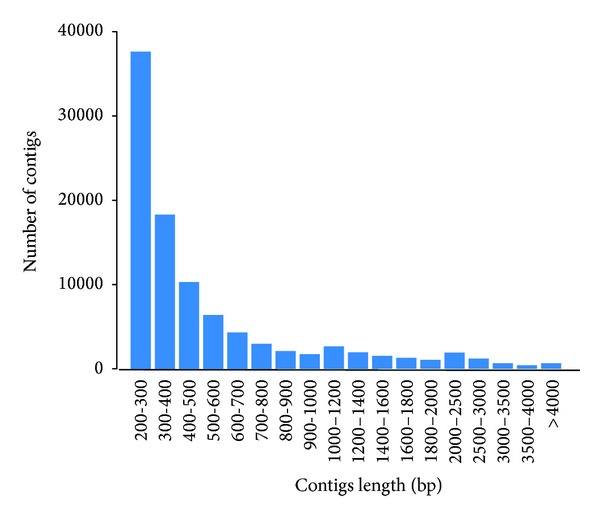
Length distribution of contigs.

Approximately 20.1 million and 22.4 million reads were obtained from male library and female library, respectively. After quality filtering, about 98.1% read of male and 99.0% read of female remain to be qualified for assembling. Sequencing saturation distribution (see Figure S1 in Supplementary Material available online at http://dx.doi.org/10.1155/2014/291067) and genes coverage statistic (Figure S2) analysis justified a deep sequencing coverage sufficient for the quantitative analysis of gene expression profiles. The clean reads were jointly assembled into 97,233 contigs with N50 of 809 bp and average length of 603 bp. The size distribution of assembled contigs was presented in Figure 1. Mapped to protein database, nearly 22.31% (21,697) of contigs were matched with known existing protein (Table S1). The function of the predicted proteins was classified with GO assignments statistically analyzed in Figure S3. In total, there were 20,582 proteins assigned with 132,920 GO terms and the three corresponding organizing principles. About 21 categories of biological process were assigned for 49,204 contigs, 11 categories of cellular component were assigned for 49,253 contigs, and 25 categories of molecular function were assigned for 41,788 contigs. Most of the cellular component genes were associated with cells and intracellular components, and most of the molecular function genes were associated with binding and catalytic activity. Mapped to reference canonical pathways in the KEGG database, 11,936 contigs were assigned with KO numbers. Through the KO number of the predicted protein, 328 metabolic pathways were constructed with various degrees (Table S1_KEGG pathway). Figure S4 showed the category distribution of biological pathways, in which the most enriched pathway is associated with signal transduction.

### 3.2. Sex-Biased Genes

Total 10348 male-biased and 3296 female-biased contigs were identified, respectively, (Table S2) showing significant expression difference between male and female. Among these sex-biased contigs, 887 (8.57%) male-biased contigs and 2193 (66.54%) female-biased contigs were annotated with known genes. In addition, 2111 contigs were identified to be expressed specifically in male and 75 contigs were identified to be expressed specifically in female. The statistical overview of transcriptome analysis was shown in Table 1. By using four strategies, sex-related well-documented genes were identified. In strategy i, we searched for well-known candidate genes already characterized to be sexually dimorphic in flounder. Among these genes, SRY-box containing protein 9 gene (*sox9*),* sox8a* mullerian inhibiting substance (*mis*), doublesex and mab-3 related transcription factor 1 gene (*dmrt1*), and so forth were predominantly expressed in male library, whereas P450 aromatase gene (*cyp19a)*, forkhead transcription factor L2 gene (*foxl2*), orphan nuclear receptor* dax1,* and so forth were obviously overexpressed in the female library. We also found out* sox6b* gene containing SRY-box, which is not reported in fish.In strategy ii, a set of keywords, including male, female, sex, sperm, egg, ovary, testis, estrogen, and androgen, were used to search sex-related genes based on annotation results. Amounts of genes consisting of the above keywords such as zona pellucida sperm-binding protein gene (*zp*), egg envelope glycoprotein-like precursor, and ovarian cancer-associated gene 2 (*ovca2*) exhibit sex-biased expression pattern. In strategy iii, among the steroidogenic enzyme genes, steroidogenic acute regulatory protein gene (*star*), 17-beta-hydroxysteroid dehydrogenase type 1 gene (*hsd17b1*), and estradiol 17-beta-dehydrogenase 12 gene (*hsd17b12*) present sex differential expression profile. In strategy iv, cathepsins (*ctss*), ropporin-1-like protein gene (*ropn1l*), ZPA domain containing protein precursor gene (*zpa*),* zpc5*, zygote arrest protein 1 gene (*zar1*), wee1-like protein kinase 2 gene (*wee2*), P43 5S RNA-binding protein gene (*42sp43*), histone H2Ax gene (h2ax), and so forth display differential expression profile between male and female. The sex-related genes identified were listed in Table 2.

### 3.3. Sexual Dimorphic Biological Pathway

**Figure 2:**
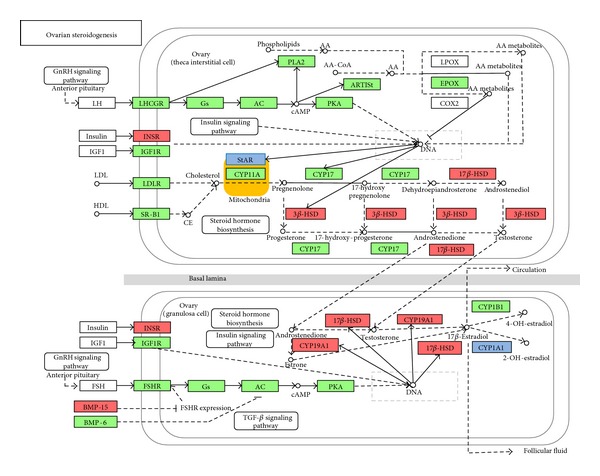
Ovarian steroidogenesis pathway. Green background indicates genes identified in flounder; red background indicates female-biased genes; blue background indicates male-biased genes.

To identify biological pathway that shows sexual dimorphism in flounder, the number of sex-biased genes was counted in different category of pathways. The result exhibits in Figure S5. It shows that the number of upregulated genes in most of metabolic pathways is much more in female than that in male. There are the most sex-biased genes in the signal transduction pathway compared with other pathways. And the significantly divergent pathways of male and female gene numbers mainly include lipid metabolism, signal transduction, translation, and cell growth death. Among them, several pathways associated with gonadal development and sex maintenance were found, such as ovarian steroidogenesis (shown as Figure 2 and Table 3), estrogen signaling pathway, progesterone-mediated oocyte maturation, prolactin signaling pathway, GnRH signaling pathway, oocyte meiosis, TGF-beta signaling pathway, steroid hormone biosynthesis, and Wnt signaling pathway.

## 4. Discussion

As a marine economic flatfish, flounder exhibit GSD + EE sex determination form, and GSD function is necessary for us to probe into. Now, little evidence can demonstrate the molecular mechanism of flounder sex determination; therefore more sex-related genes and explicit biological pathways are needed to imply the mechanism. In this study, we used RNA-seq technology to identify large quantities of sex-biased genes and illustrated its function from the perspective of biological pathways. Among these sex-biased genes, some are associated with the sex determination and gonadal development as reported. The other sex-biased genes need further study to investigate their connection with sex determination and differentiation. The sex-related genes identified in this study could provide an important clue for sex determination mechanism of flounder. In addition, the novel contigs may be from unknown gene sequence or alternatively spliced transcripts. Yano et al. [8] characterized a novel gene expressed only in testis through analyzing gonadal transcriptome of rainbow trout (*O. mykiss*), and further study revealed that this gene is Y chromosome sequence tightly linked with sex locus and necessary to trigger testicular differentiation. Therefore, our study also provides considerable novel sex-biased genes for further study on sex determination of flounder.

### 4.1. Sex Differences in Gene Expression Profiles of Gonads

We analyzed the overall gene expression profiles of gonads and identified numerous sex-related genes. Some of the sex-biased genes are known to show sexual dimorphism between ovary and testis testifying the reliability of the selection criterion in the results. Our analyses also found considerable previously uncharacterized sex-biased genes. Further functional characterization of these genes using transgenic overexpression, knockout strategies, and knockdown strategies may help elucidate the molecular mechanisms controlling sex determination and gonadal development in teleost.

#### 4.1.1. Male-Biased Genes

In the present study, more contigs were found to be male-biased genes than female-biased genes. For lack of genomic sequence of flounder, a large amount of male-biased contigs cannot be further assembled and annotated. Among the annotated genes, several well-documented and important male-enriched genes are listed in more detail.* Dmrt1* is expressed in the embryonic gonads of many vertebrates. It was thought to repress the female pathway through inhibition of* cyp19a1a* expression in Nile tilapia (*O. niloticus*) [34]. In medaka (*O. latipes*),* dmy*, a duplicated copy of the* dmrt1* on the Y chromosome, has been confirmed as sex determining gene [5, 6]. Our result shows the male-dominant expression of* dmrt1* in flounder as reported [25]. These reveal that* dmrt1* may be an important factor for flounder testicular differentiation. Three members of sox gene family including* sox9*,* sox8a*, and* sox6b* were identified to be male predominantly expressed genes. In teleost,* sox9* has been associated with testicular development, with* sox9*-expressing cells differentiating into Sertoli cells [35]. Thestudy on* sox8a *was only reported in orange-spotted grouper (*Epinephelus coioides)*, and it was expressed in diverse tissues and increased during testicular developmental stages [36]. No document covered the function of* sox6b*. AMY-1-associating protein was found to be associated with protein kinase A anchor protein 84/149 in the mitochondria in human sperm, suggesting that it plays a role in spermatogenesis [37]. Heat shock protein 90 (HSP90) interacts with steroid hormone receptors, signaling kinases, and various transcription factors [38]. However, the mechanism by which HSP90 interacts with different proteins in various pathways remains unclear. Here in our study, we find *hsp 90* *α* genehighly expressed in both ovary and testis tissues, while *hsp 90* *β* gene exhibited upregulated expression pattern in testis. Several proteins associated with sperm mobility show absolute predominance in testis. In human, Ropporin-1-like protein is spermatogenic cell-specific protein that serves as an anchoring protein for the A-kinase anchoring protein, which may function as a regulator of both motility- and head-associated functions such as capacitation and the acrosome reaction [39]. Sperm flagellar protein 2 plays an important role in spermatogenesis and flagellar assembly. And a loss of function mutation in* spef2* of mice caused the big giant head phenotype [40]. Axonemal dynein light intermediate polypeptide may take part in the formation of sperm flagella and play a dynamic role in flagellar motility. As is well known, both cytochrome c oxidase subunit I and ATP synthase F0 subunit 6 are components of the respiratory chain supplying energy for sperm mobility.

#### 4.1.2. Female-Biased Genes

Ovary is the female reproductive system and ovum-producing reproductive organ. It is responsible for the synthesis of estrogen and oogenesis. More and more female-biased genes were found to play important roles in the above processes. In this study, nearly 66.5% of female-biased genes were annotated, of which well-documented ovary markers such as* cyp19a*,* foxl2*,* zp*, cathepsins, and* 42sp43* were identified as female-biased genes.* Cyp19a* is responsible for encoding P450 aromatase, critical enzyme catalyzing the process of transforming androgen into estrogen [41]. In teleost,* cyp19a* has been proved to play an important role in sex differentiation and ovarian development, and it is regarded as a reliable early marker of ovarian differentiation [42]. In addition,* foxl2*, encoding the activating transcription factor of* cyp19a*, has been identified to be uniquely expressed in female, which may be one reason for the higher expression level of* cyp19a *in female than in male justified by Yamaguchi et al. [24]. In mammal,* zar1* gene is oocyte-specific maternal-effect gene that functions at the oocyte-to-embryo transition [43]. Wee1-like protein kinase 2 is oocyte-specific protein tyrosine kinase that phosphorylates and inhibits cyclin-dependent kinase 1 (CDK1) and acts as a key regulator of meiosis in* Xenopus* [44]. Histone* H2Ax* is reported to play an important role in chromatin remodeling and associated silencing in male mouse meiosis [45]. However, we found* h2ax *highly expressed in ovary rather than testes in our result, which suggests that it may act on oogenesis of female flounder. The developing oocyte is surrounded by an acellular envelope that is composed of zona pellucida proteins. It is reported that* zpa*,* zpc*, and* zp3* genes and these proteins participate in taxon-specific sperm-egg binding during fertilization process and protect embryo at early developmental stage in mammals [46].* Zps* were also identified as female-biased genes in this study. Vitellogenesis is the principal event responsible for the enormous growth of oocytes in many teleosts, during which most nutritive products are taken up and stored for developing embryo. Vitellogenin receptor is involved in uptake of vitellogenin by endocytosis. Enzymes such as cathepsins are responsible for the degradation of vitellogenin into yolk protein for storage in the oocyte [47]. P43 5S RNA-binding protein is combined with 5S rRNA to comprise 42S ribonucleoprotein storage particle. In addition, transcription factor IIIA acts as both a positive transcription factor for 5S RNA genes and a specific RNA-binding protein that complex with 5S RNA in oocytes to form the 7S ribonucleoprotein storage particle [48]. According to Diaz De Cerio et al., 5 S RNA and associate protein could constitute a sensitive and universal marker of oogenesis and oocyte differentiation in fish [49].

### 4.2. Sex Steroids and Biosynthetic Pathway

Gonad is an important organ that is responsible for producing gametes and sex hormones. Steroid hormones are small, hydrophobic hormones that can permeate membranes and therefore can bind to specific nuclear receptors, including estrogen receptors (ER) and androgen [50]. It can form a complex with a hormone receptor and enter the nucleus where this complex can bind to DNA and result in the translation of specific mRNA and proteins. These can give rise to specific physiological responses including sex differentiation and germ-cell development [51]. In the present study, 20 genes were identified to be involved in flounder ovarian steroidogenesis pathway. In this pathway, all steroids are synthesized from cholesterol, whose transportation from intracellular source into the mitochondria is the rate-limiting step in steroidogenesis [52]. This transmembrane transport process is facilitated by the steroidogenic acute regulatory protein [53]. And the present study identified* star* as male-biased gene.

Male hormone testosterone is formed from pregnenolone by two pathways, delta5 pathway via dehydroepiandrosterone and delta4 pathway via androstenedione. The enzyme P450c17 is responsible for the 17,20-lyase and 17-alpha-hydroxylase activities in respective pathways. Two forms of P450c17 were identified in some teleost, which were P450c17-I and P450c17-II, encoded by* cyp17-I* and* cyp17-II* genes, respectively [54]. P450c17-I possesses both hydroxylase and lyase activities, while P450c17-II only has hydroxylase activity. In medaka, P450c17-I is essential for the production of estradiol-17*β* (E2) during oocyte growth, while P450c17-II plays a vital role in production of 17*α*, 20*β*-dihydroxy-4-pregnen-3-one (17*α*, 20*β*-DP) during oocyte maturation [55]. In our study, the transcripts of* cyp17-II* were only found. According to Ding et al., the variation trends of T and E2 level were consistent with the* cyp17-II* expression pattern in flounder ovary [26]. Nevertheless,* cyp19a* still plays a central role in the synthesis of E2. The suppression of* cyp19a* expression at male-inducing temperature leads to masculinization, which further supports the importance of this enzyme and its product (estrogen) in flounder ovarian differentiation [23]. Additionally, 17-beta-hydroxysteroid dehydrogenase (*hsd17b*) and 3-beta-hydroxysteroid dehydrogenase (*hsd3b*) were identified to be female predominantly expressed genes, which indicate their important role in synthesis of estrogen.

The steroidogenic enzymes expression profile may help us to demonstrate the difference of steroid level between male and female. Based on our previous study, both testosterone and estradiol-17*β* (E2) levels are on rise from ovarian developmental stage I to stage IV. The decrease of E2 level is detected in temperature-induced masculinized groups compared to control groups [56]. During testicular developmental stage, E2 in the serum stay in low level while T level varies significantly during different developmental stage. The variation of sex steroids level may be tightly linked with gonadal development and maturation of germ cells.

## 5. Conclusion

This is the first report of flounder gonadal transcriptome using RNA-seq technology. We generated a large number of ESTs collection and identified numerous differentially expressed genes between ovary and testis. According to annotation information, sex-related biological pathways including ovarian steroidogenesis were found. The dimorphic expression of overall sex-related genes provides further insights into sexual difference and gonadal development. Our result also provides an archive for further study on molecular mechanism underlying sex determination.

## Supplementary Material

Table S1: The list of genes annotation information and KEGG pathway.
Table S2: The list of sex-biased genes. Notes, Afemale*；*Bmale*；* log2*（*fold_change*）*, log2(RPKM XX/RPKM XY).
Figure S1: Sequencing saturation curve. Horizontal coordinate stands for read number. Vertical coordinate stands for gene number. When the read number exceeds 20 million, the gene number detected is approaching saturation.
Figure S2: Genes coverage statistic pie chart. It demonstrates that the gene coverage of both male and female is above 90% and reaching summit.
Figure S3: Gene Ontology (GO) assignment class. A, molecular function; B, cellular component; C, biological process. Horizontal coordinate stands for GO secondary term; Vertical coordinate stands for gene number subjected to the GO term.
Figure S4: Biological pathway class distribution. A, Genetic Information Processing; B, Organismal Systems; C, Cellular Processes; D, Environmental Processing; E, Metabolism.
Figure S5: Sexual dimorphic biological pathway. Horizontal coordinate stands for the number of up-regulated genes; Vertical coordinate stands for biological pathway.

## Figures and Tables

**Table 1:** Overview of transcriptome analysis.

Total number of reads	42,640,333
Number of male reads	20,161,959
Number of female reads	22,478,374
Number of male reads in contigs	14,266,081
Number of female reads in contigs	20,939,047
Number of contigs from assembly	97,233
Number of annonated contigs	21,697
Number of contigs >400 bp	41,135
Number of male only contigs	25,226
Number of female only contigs	10,750
Number of mixed contigs	61,257
Sex-biased genes with *q*-value ≤ 1 × 10^−3^ AND |log_2_⁡ ratio| ≥ 2	13,644
Male-biased contigs	10,348
Annonated male-biased genes	887
Female-biased contigs	3296
Annonated female-biased genes	2193

**Table 2:** Representative sex-biased genes involved in sex determination and development.

Contigs	Length	Function	log_2_⁡ (fold)	*q*-value	Sex	Gene
Comp134314_c0_seq1	2117	P450 aromatase	8.1274	1.08*E* − 71	Female	*cyp19a *
Comp128200_c0_seq1	1721	Forkhead transcription factor L2	7.7734	1.51*E* − 29	Female	*foxl2 *
Comp129336_c0_seq1	697	Orphan nuclear receptor Dax1	2.3341	2.96*E* − 07	Female	*dax1 *
Comp135979_c0_seq1	3970	Vitellogenin receptor	2.4058	0	Female	*vtgr *
Comp131376_c0_seq1	1862	Zona pellucida sperm-binding protein 4	6.733436	0	Female	*zp4 *
Comp129690_c1_seq1	1123	Zona pellucid sperm-binding protein 3	6.8619	0	Female	*zp3 *
Comp126058_c0_seq1	1449	Zona pellucida sperm-binding protein	6.1157	0	Female	*zp *
Comp96429_c0_seq1	668	Histone H2A.x	6.3287	0	Female	*h2a.x *
Comp124668_c0_seq1	1372	Zygote arrest protein 1	6.556118	0	Female	*zar1 *
Comp138502_c2_seq1	2515	ZPA domain containing protein precursor	7.7351	0	Female	*zpa *
Comp131365_c0_seq1	2442	ZPC5	6.5016	0	Female	*zpc5 *
Comp130117_c0_seq1	1092	High choriolytic enzyme 2	7.5466	0	Female	*hce2 *
Comp135460_c1_seq1	4433	Protein fem-1 homolog C	2.2905	1.58*E* − 173	Female	*fem1c *
Comp130482_c0_seq1	1936	Frizzled-3	3.2588	7.16*E* − 20	Female	*fzd3 *
Comp126332_c0_seq1	1789	P43 5S RNA-binding protein	8.3272	0	Female	*42sp43 *
Comp134412_c0_seq1	3026	Transcription factor IIIA	3.7385	0	Female	*gtf3a *
Comp129656_c0_seq1	2020	Wee1-like protein kinase 2	6.9006	0	Female	*wee2 *
Comp126733_c0_seq1	1431	Cathepsin S precursor	5.1171	0	Female	*ctss *
Comp137073_c0_seq1	2247	G2/mitotic-specific cyclin-B1-like	6.7561	0	Female	*ccnb1 *
Comp126534_c0_seq1	1128	Ovarian cancer-associated gene 2 protein	2.744	4.03*E* − 57	Female	*ovca2 *
Comp130477_c0_seq1	1312	17-beta-Hydroxysteroid dehydrogenase type 1	5.2759	3.24*E* − 34	Female	*hsd17b1 *
Comp128629_c0_seq1	1286	SRY-box containing protein 9	−4.0548	3.25*E* − 33	Male	*sox9 *
Comp138102_c0_seq1	1849	Mullerian inhibiting substance	−5.7273	1.34*E* − 283	Male	*mis *
Comp135961_c0_seq1	2582	Sox8a	−4.3658	0	Male	*sox8a *
Comp125855_c0_seq1	1045	Steroidogenic acute regulatory protein	−2.1114	6.75*E* − 10	Male	*star *
Comp70046_c1_seq1	523	SRY-box containing gene 6b	−2.9959	2.38*E* − 25	Male	*sox6b *
Comp125855_c0_seq1	1045	Steroidogenic acute regulatory protein	−2.1114	6.75*E* − 10	Male	*star *
Comp70129_c0_seq1	1376	Cytochrome c oxidase subunit I	−13.8122	0	Male	*cox I *
Comp127682_c0_seq1	960	ATP synthase F0 subunit 6	−12.5508	4.52*E* − 268	Male	*atp5g *
Comp127263_c1_seq1	1000	Heat shock protein 90 alpha	−6.5865	0	Male	*hsp 90* *α*
Comp132152_c1_seq1	3496	Sperm flagellar protein 2	−3.2844	3.69*E* − 302	Male	*spef *
Comp138413_c1_seq1	2470	AMY-1-associating protein	−2.2291	9.22*E* − 53	Male	*aat1 *
Comp131350_c0_seq1	1398	Axonemal dynein light intermediate polypeptide 1	−9.422	0	Male	*dnali1 *
Comp128419_c1_seq1	659	Ropporin-1-like protein	−8.2764	2.46*E* − 27	Male	*ropn1l *

Note: Fold indicates RPKM (female)/RPKM (male); *q*-value indicates false discove rate (FDR).

**Table 3:** Genes identified to take part in the ovarian steroidogenesis pathway.

Contigs	Gene function	Protein	*e*-value	log⁡ (fold)
Comp1153048_c0_seq1	Luteinizing hormone receptor	LHR	3*E* − 45	NA/female
Comp67657_c0_seq1	Guanine nucleotide-binding protein G(s) subunit alpha	Gs	1*E* − 51	0.299
Comp127442_c3_seq1	Insulin receptor	INSR	1*E* − 126	2.911
Comp123852_c0_seq1	Low-density lipoprotein receptor-related protein 1	LDLR	0	1.607
Comp137524_c1_seq1	Follicle stimulating hormone receptor II	FSHR	0	−1.703
Comp108327_c0_seq1	cAMP-dependent protein kinase catalytic subunit PRKX	PKA	8*E* − 74	1.911
Comp138426_c0_seq1	Acyl-protein thioesterase 2	ACOT2	1*E* − 104	1.514
Comp1185767_c0_seq1	Cholesterol side chain cleavage cytochrome P450	CYP11A1	8*E* − 66	NA/male
Comp945537_c0_seq1	17-alpha-Hydroxylase	CYP17	9*E* − 93	1.446
Comp125855_c0_seq1	Steroidogenic acute regulatory protein, mitochondrial	StAR	1*E* − 65	−2.111
Comp134314_c0_seq1	P450 aromatase	CYP19A1	0	8.127
Comp123514_c0_seq1	3-beta-Hydroxysteroid dehydrogenase type II	HSD3b	2*E* − 94	2.288
Comp134625_c0_seq1	Estradiol 17-beta-dehydrogenase 12-A	HSD17b	1*E* − 168	4.625
Comp131665_c2_seq1	Cytochrome P450 1B1	CYP1B1	0	−1.964
Comp123774_c0_seq1	Cytochrome P450 1A	CYP1A1	0	−2.691
Comp126639_c0_seq1	Catechol O-methyltransferase	COMT	1*E* − 134	−1.249
Comp507499_c0_seq1	Cytosolic phospholipase A2	CPLA2	3*E* − 68	−2.548
Comp80365_c0_seq1	Cytochrome P450 2J2	CYP2J	1*E* − 116	0.451
Comp133304_c0_seq1	Scavenger receptor class B member	SCARB1	0	0.919
Comp136599_c2_seq1	Adenylate cyclase type 1	ADCY1	3*E* − 25	4.797

Note: NA indicates solely identification in female or male; fold indicates RPKM (female)/RPKM (male).
