# Supplementary figures and images for: Gonadal Transcriptome Analysis of Male and Female Olive Flounder (Paralichthys olivaceus)
Source: Biomed Res Int. 2014 Jul 6;2014:291067. doi: 10.1155/2014/291067 (PMC4121151; doi:10.1155/2014/291067)

# 115YF-L

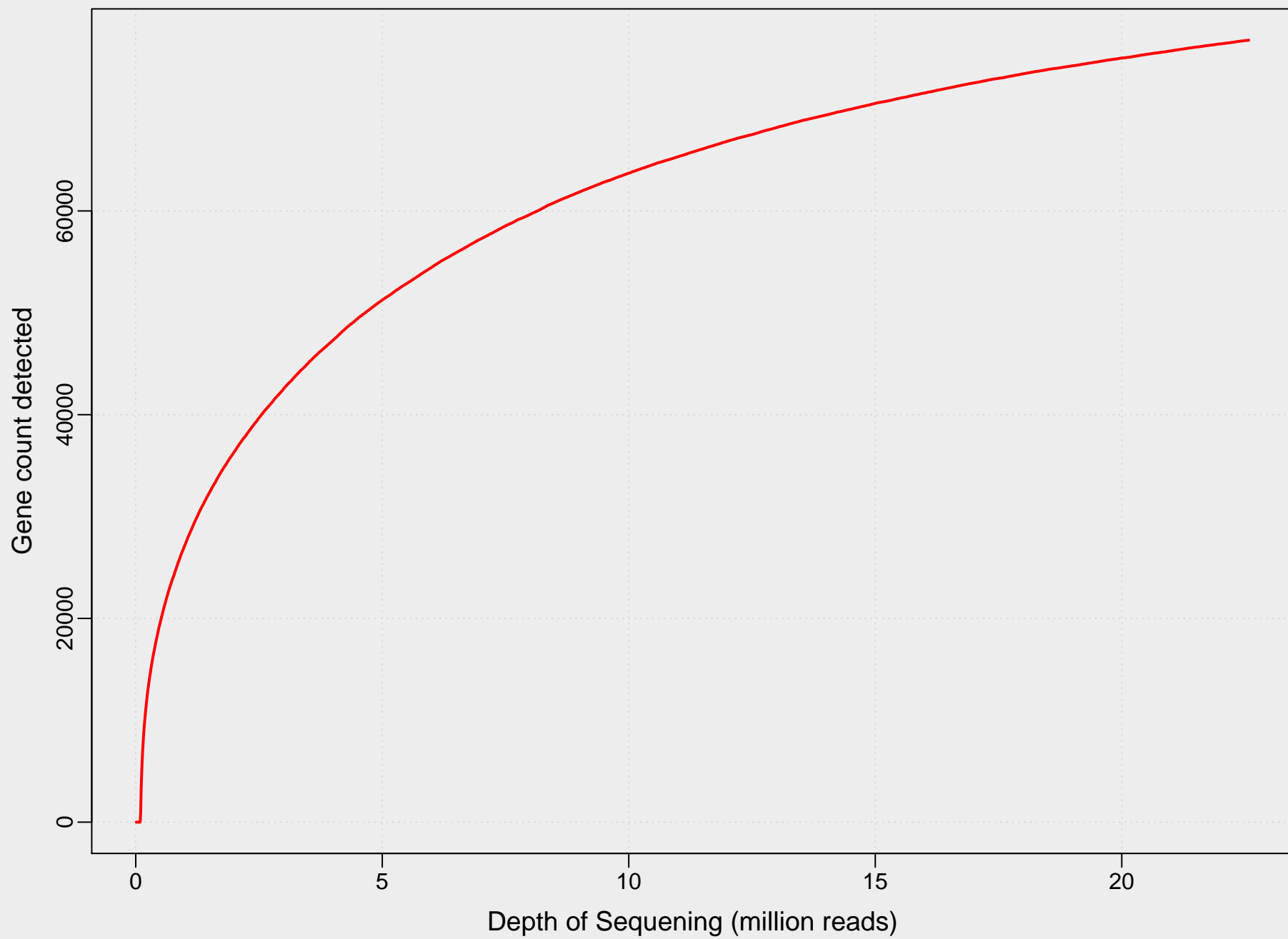

YF-J

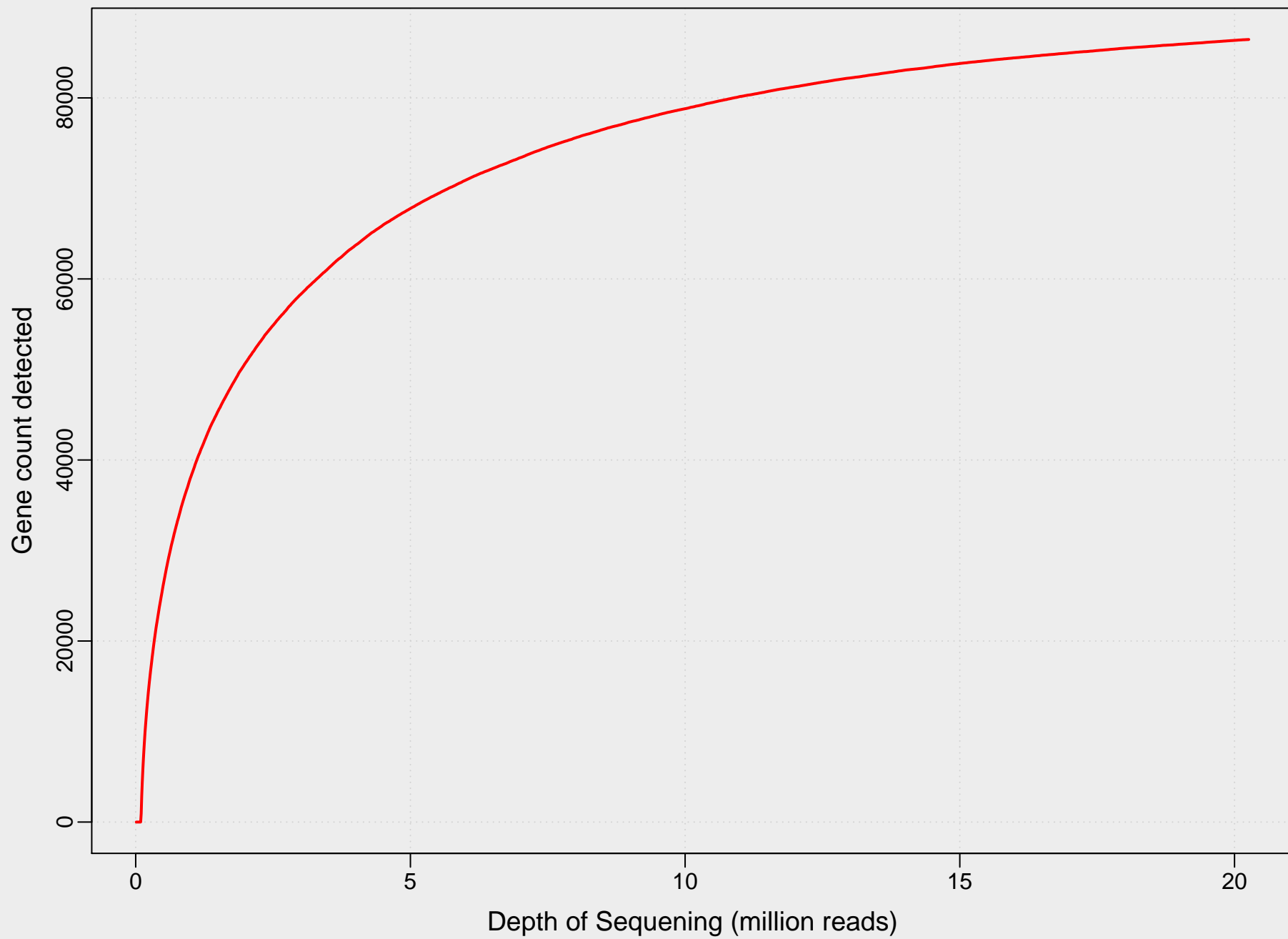

Supplement: Supplementary file 1 — Table S1: The list of genes annotation information and KEGG pathway. Table S2: The list of sex-biased genes. Notes, Afemale；Bmale； log2（fold_change）, log2(RPKM XX/RPKM XY). Figure S1: Sequencing saturation curve. Horizontal coordinate stands for read number. Vertical coordinate stands for gene number. When the read number exceeds 20 million, the gene number detected is approaching saturation. Figure S2: Genes coverage statistic pie chart. It demonstrates that the gene coverage of both male and female is above 90% and reaching summit. Figure S3: Gene Ontology (GO) assignment class. A, molecular function; B, cellular component; C, biological process. Horizontal coordinate stands for GO secondary term; Vertical coordinate stands for gene number subjected to the GO term. Figure S4: Biological pathway class distribution. A, Genetic Information Processing; B, Organismal Systems; C, Cellular Processes; D, Environmental Processing; E, Metabolism. Figure S5: Sexual dimorphic biological pathway. Horizontal coordinate stands for the number of up-regulated genes; Vertical coordinate stands for biological pathway. [file 291067.f1.zip › Figure S1.pdf]

# 115YF-L\_percentile of gene coverage

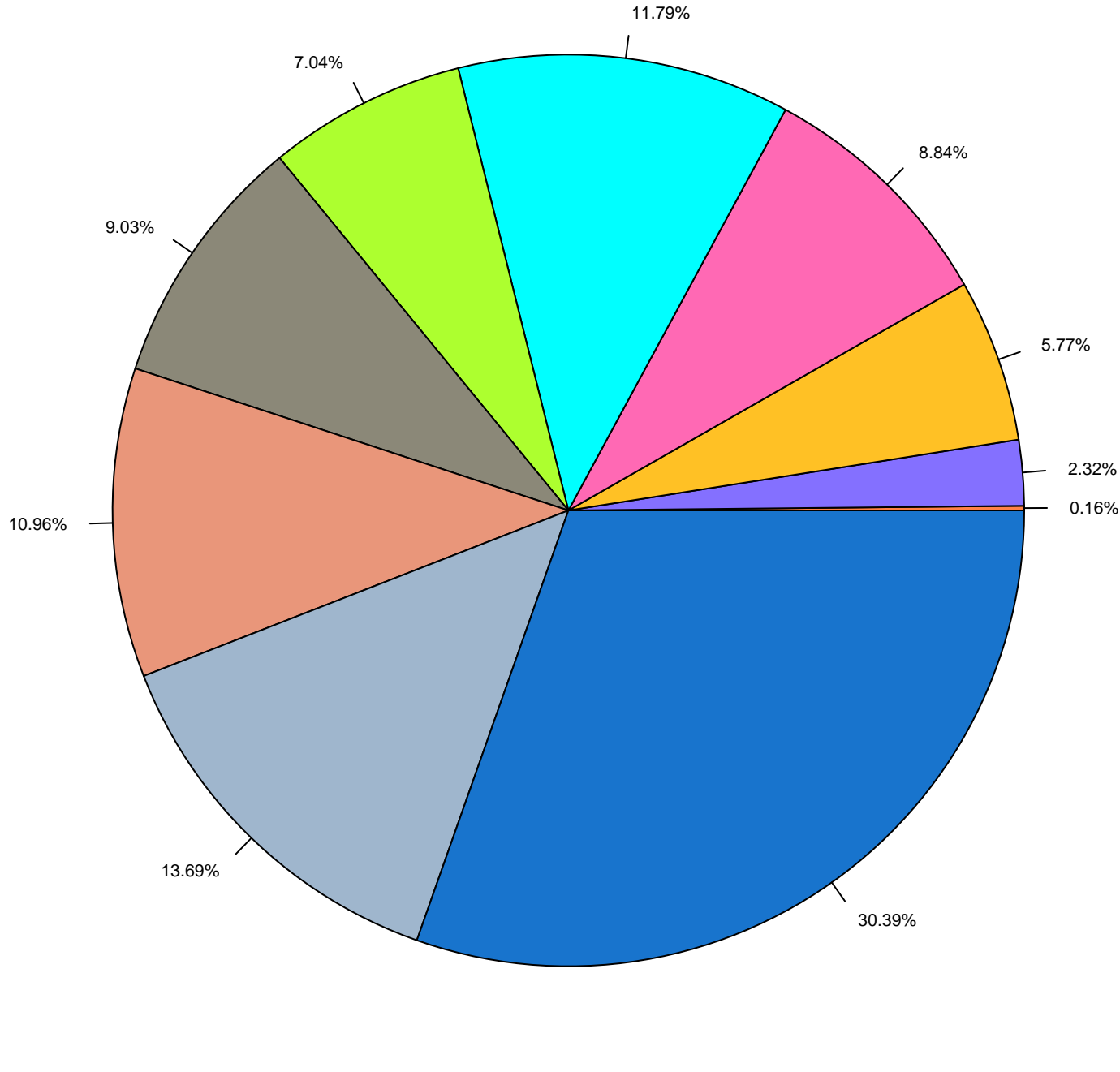

# YF-J\_percentile of gene coverage

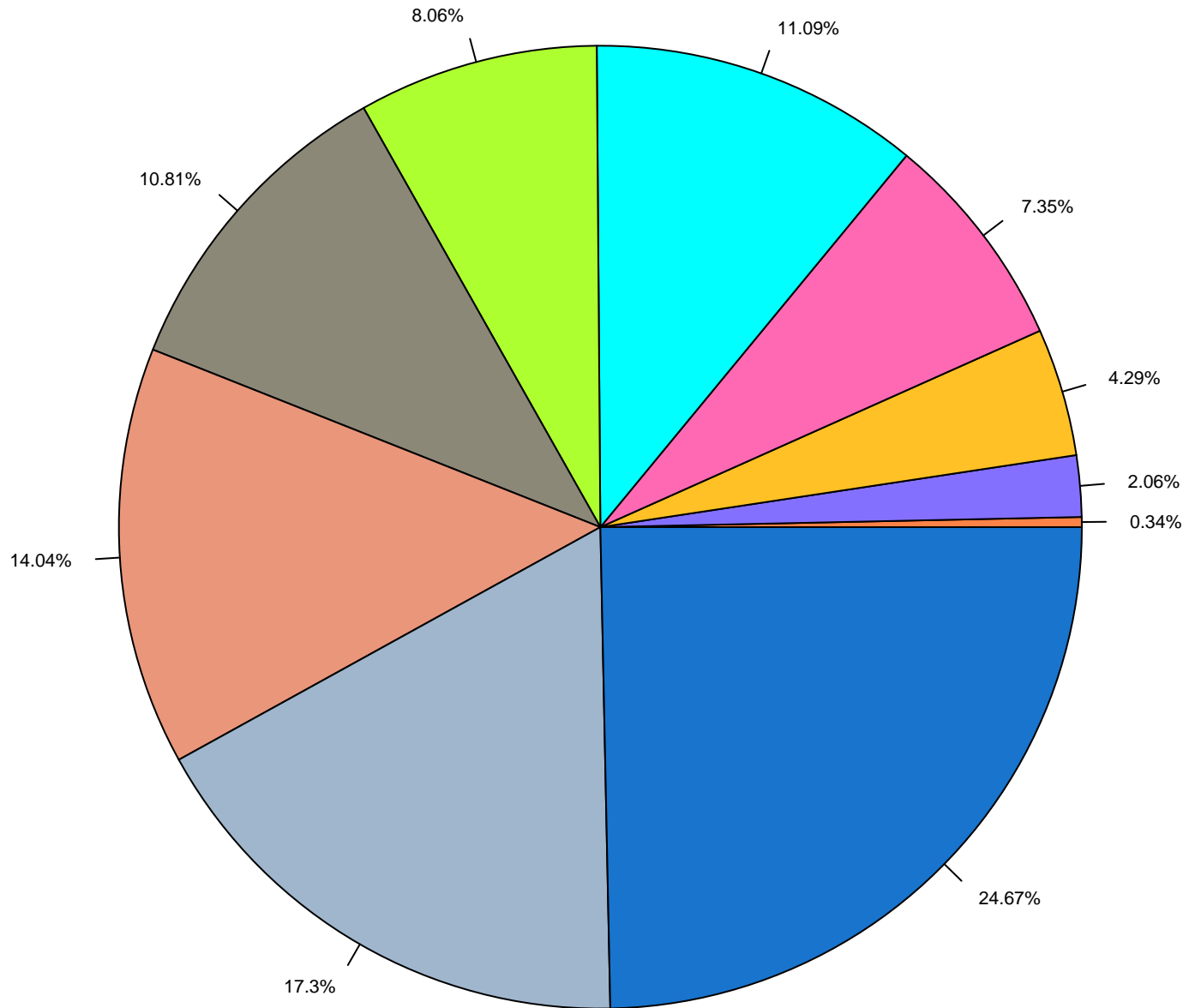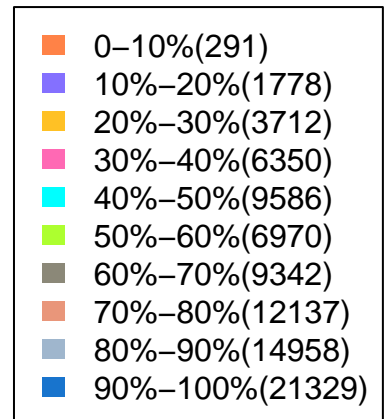

Supplement: Supplementary file 1 — Table S1: The list of genes annotation information and KEGG pathway. Table S2: The list of sex-biased genes. Notes, Afemale；Bmale； log2（fold_change）, log2(RPKM XX/RPKM XY). Figure S1: Sequencing saturation curve. Horizontal coordinate stands for read number. Vertical coordinate stands for gene number. When the read number exceeds 20 million, the gene number detected is approaching saturation. Figure S2: Genes coverage statistic pie chart. It demonstrates that the gene coverage of both male and female is above 90% and reaching summit. Figure S3: Gene Ontology (GO) assignment class. A, molecular function; B, cellular component; C, biological process. Horizontal coordinate stands for GO secondary term; Vertical coordinate stands for gene number subjected to the GO term. Figure S4: Biological pathway class distribution. A, Genetic Information Processing; B, Organismal Systems; C, Cellular Processes; D, Environmental Processing; E, Metabolism. Figure S5: Sexual dimorphic biological pathway. Horizontal coordinate stands for the number of up-regulated genes; Vertical coordinate stands for biological pathway. [file 291067.f1.zip › Figure S2.pdf]

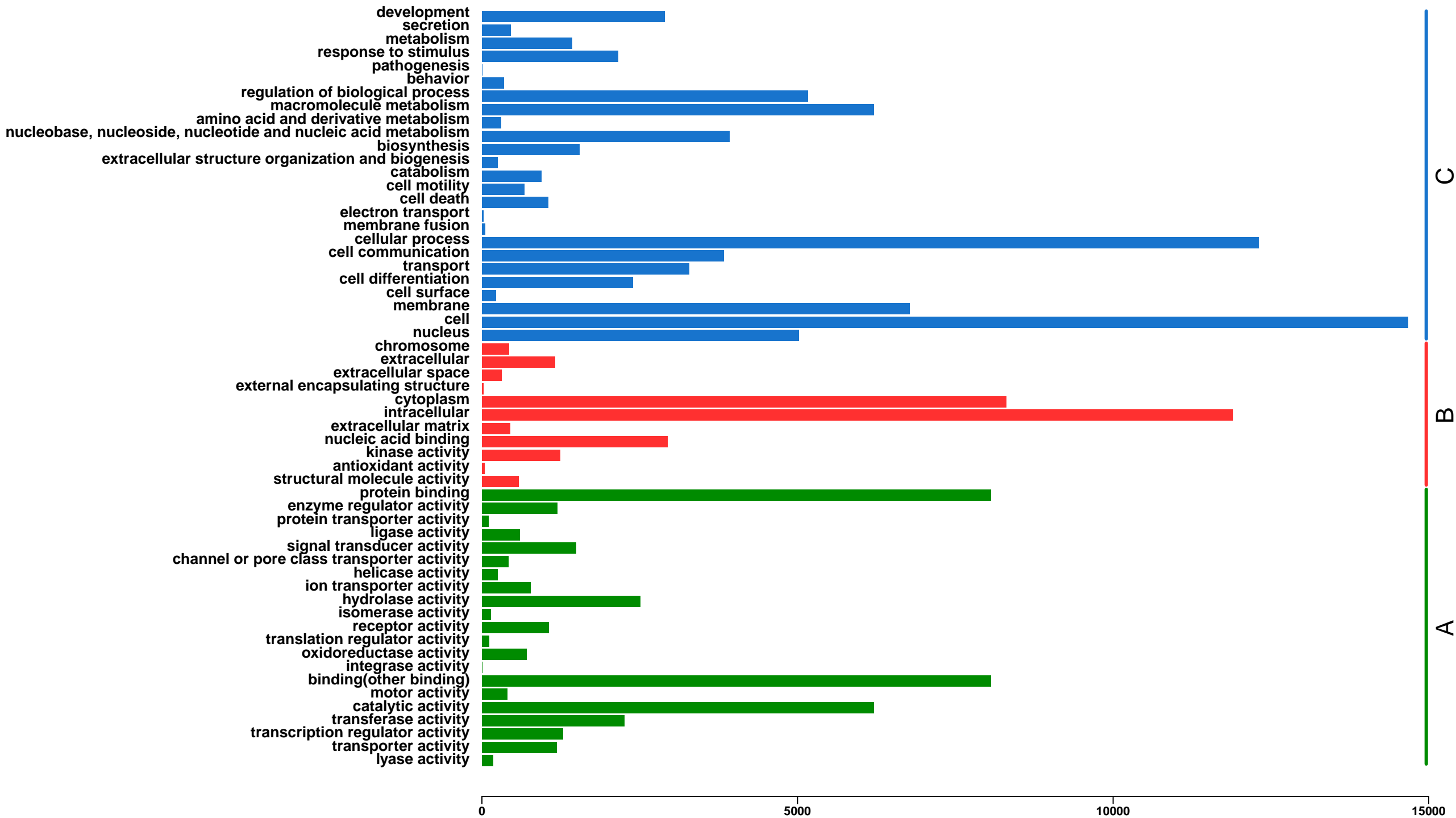

Supplement: Supplementary file 1 — Table S1: The list of genes annotation information and KEGG pathway. Table S2: The list of sex-biased genes. Notes, Afemale；Bmale； log2（fold_change）, log2(RPKM XX/RPKM XY). Figure S1: Sequencing saturation curve. Horizontal coordinate stands for read number. Vertical coordinate stands for gene number. When the read number exceeds 20 million, the gene number detected is approaching saturation. Figure S2: Genes coverage statistic pie chart. It demonstrates that the gene coverage of both male and female is above 90% and reaching summit. Figure S3: Gene Ontology (GO) assignment class. A, molecular function; B, cellular component; C, biological process. Horizontal coordinate stands for GO secondary term; Vertical coordinate stands for gene number subjected to the GO term. Figure S4: Biological pathway class distribution. A, Genetic Information Processing; B, Organismal Systems; C, Cellular Processes; D, Environmental Processing; E, Metabolism. Figure S5: Sexual dimorphic biological pathway. Horizontal coordinate stands for the number of up-regulated genes; Vertical coordinate stands for biological pathway. [file 291067.f1.zip › Figure S3.pdf]

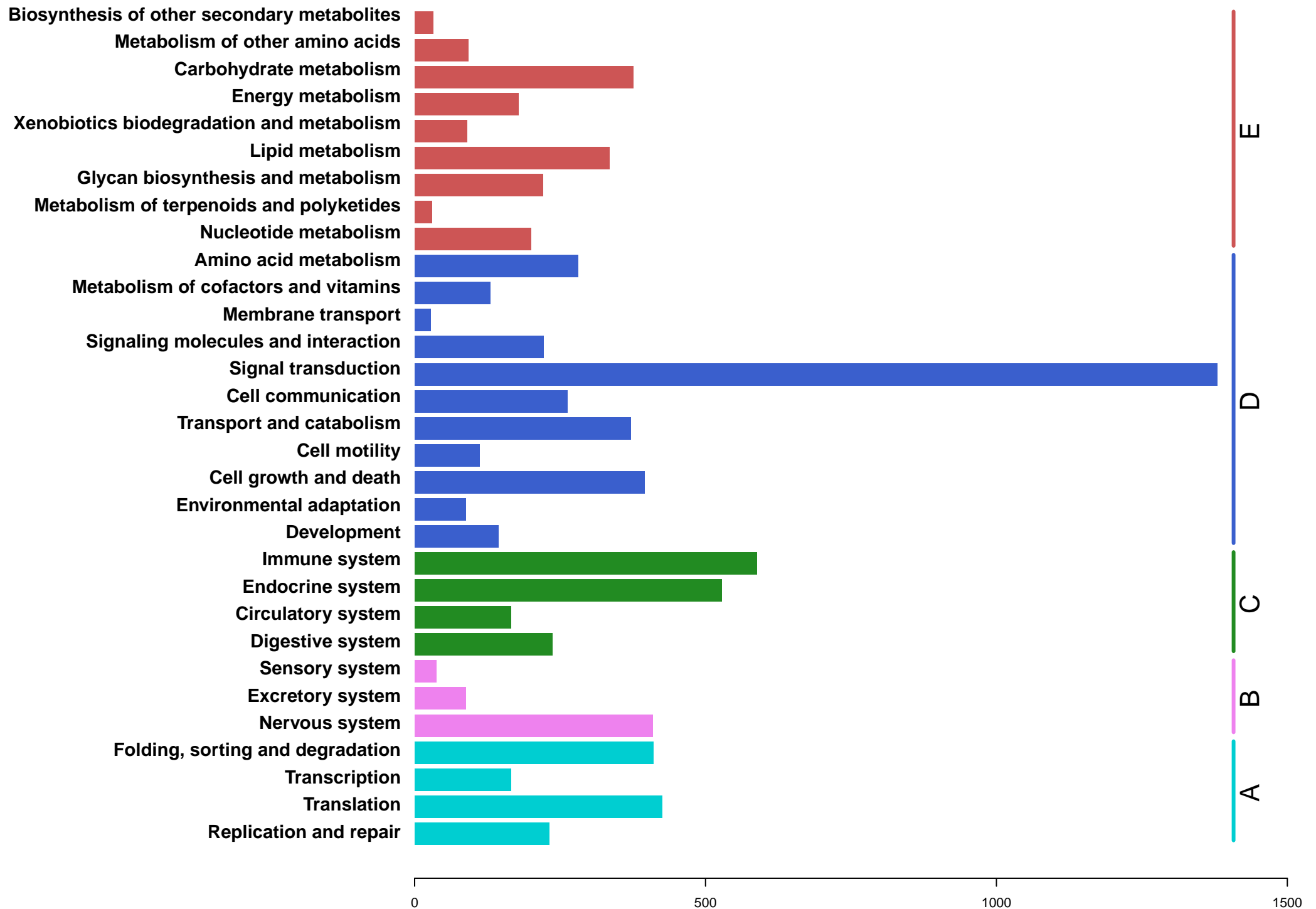

Supplement: Supplementary file 1 — Table S1: The list of genes annotation information and KEGG pathway. Table S2: The list of sex-biased genes. Notes, Afemale；Bmale； log2（fold_change）, log2(RPKM XX/RPKM XY). Figure S1: Sequencing saturation curve. Horizontal coordinate stands for read number. Vertical coordinate stands for gene number. When the read number exceeds 20 million, the gene number detected is approaching saturation. Figure S2: Genes coverage statistic pie chart. It demonstrates that the gene coverage of both male and female is above 90% and reaching summit. Figure S3: Gene Ontology (GO) assignment class. A, molecular function; B, cellular component; C, biological process. Horizontal coordinate stands for GO secondary term; Vertical coordinate stands for gene number subjected to the GO term. Figure S4: Biological pathway class distribution. A, Genetic Information Processing; B, Organismal Systems; C, Cellular Processes; D, Environmental Processing; E, Metabolism. Figure S5: Sexual dimorphic biological pathway. Horizontal coordinate stands for the number of up-regulated genes; Vertical coordinate stands for biological pathway. [file 291067.f1.zip › Figure S4.pdf]

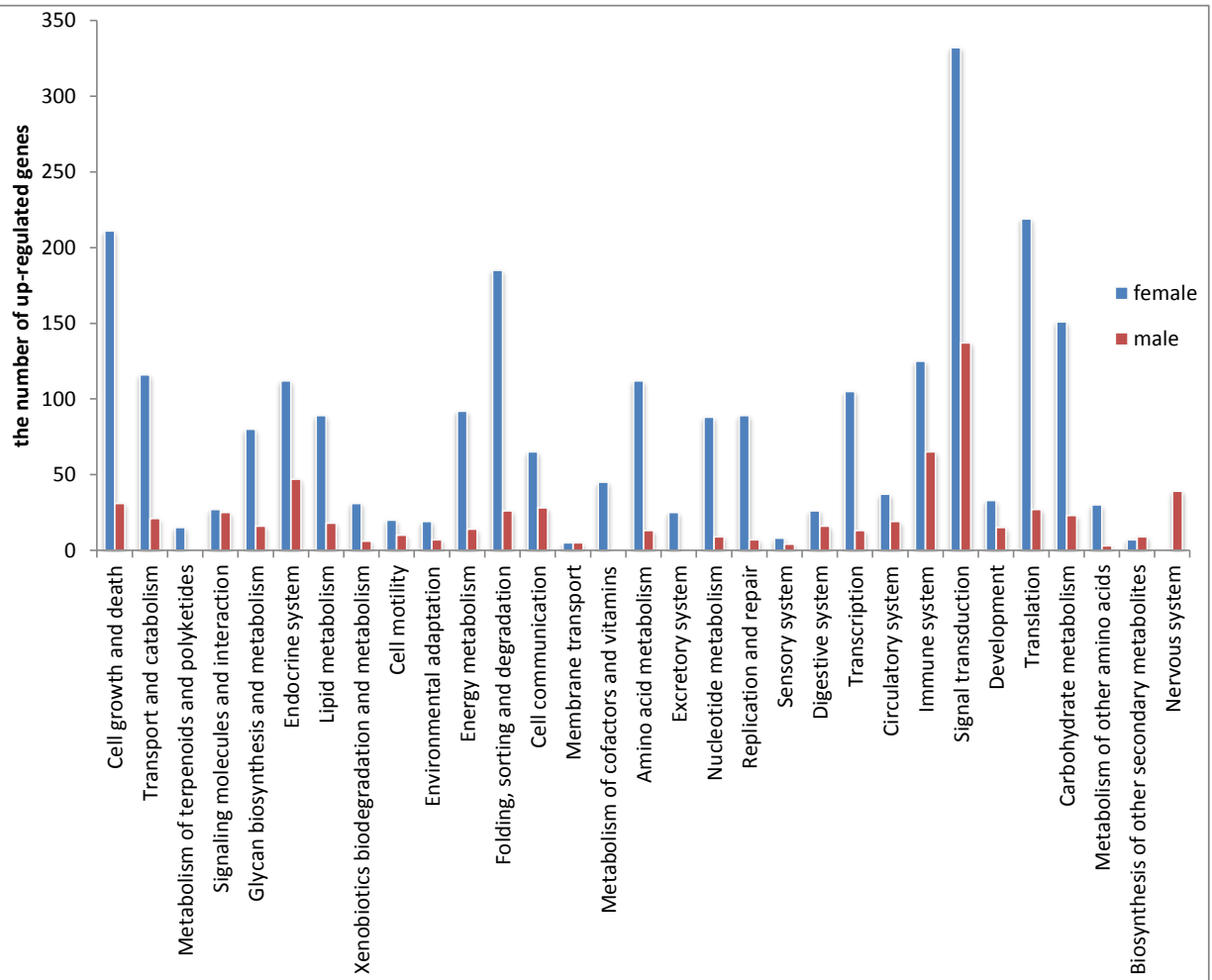

Supplement: Supplementary file 1 — Table S1: The list of genes annotation information and KEGG pathway. Table S2: The list of sex-biased genes. Notes, Afemale；Bmale； log2（fold_change）, log2(RPKM XX/RPKM XY). Figure S1: Sequencing saturation curve. Horizontal coordinate stands for read number. Vertical coordinate stands for gene number. When the read number exceeds 20 million, the gene number detected is approaching saturation. Figure S2: Genes coverage statistic pie chart. It demonstrates that the gene coverage of both male and female is above 90% and reaching summit. Figure S3: Gene Ontology (GO) assignment class. A, molecular function; B, cellular component; C, biological process. Horizontal coordinate stands for GO secondary term; Vertical coordinate stands for gene number subjected to the GO term. Figure S4: Biological pathway class distribution. A, Genetic Information Processing; B, Organismal Systems; C, Cellular Processes; D, Environmental Processing; E, Metabolism. Figure S5: Sexual dimorphic biological pathway. Horizontal coordinate stands for the number of up-regulated genes; Vertical coordinate stands for biological pathway. [file 291067.f1.zip › Figure S5.pdf]
